# Comparison of efficacy and safety of preoperative Chemoradiotherapy in locally advanced upper and middle/lower rectal cancer

**DOI:** 10.1186/s13014-018-0987-0

**Published:** 2018-03-27

**Authors:** Ming-Yii Huang, Hsin-Hua Lee, Hsiang-Lin Tsai, Ching-Wen Huang, Yung-Sung Yeh, Cheng-Jen Ma, Chun-Ming Huang, Chiao-Yun Chen, Joh-Jong Huang, Jaw-Yuan Wang

**Affiliations:** 1 Department of Radiation Oncology, Kaohsiung Medical University Hospital, Kaohsiung, Taiwan; 2 Department of Radiation Oncology, Faculty of Medicine, College of Medicine, Kaohsiung Medical University, Kaohsiung, Taiwan; 3 Division of Colorectal Surgery, Department of Surgery, Kaohsiung Medical University Hospital, Kaohsiung Medical University, No. 100 Tzyou 1st Road, Kaohsiung, 807 Taiwan; 4 Department of Surgery, Faculty of Medicine, College of Medicine, Kaohsiung Medical University, Kaohsiung, Taiwan; 5 Division of Trauma, Department of Surgery, Kaohsiung Medical University Hospital, Kaohsiung, Taiwan; 6 Department of Emergency Medicine, Kaohsiung Medical University Hospital, Kaohsiung Medical University, Kaohsiung, Taiwan; 7 Division of Digestive and General Surgery, Department of Surgery, Kaohsiung Medical University Hospital, Kaohsiung, Taiwan; 8 Department of Medical Imaging, Kaohsiung Medical University Hospital, Kaohsiung, Taiwan; 9 Department of Radiology, Faculty of Medicine, College of Medicine, Kaohsiung Medical University, Kaohsiung, Taiwan; 10 Department of Family Medicine, Kaohsiung Medical University Hospital, No. 100 Tzyou 1st Road, Kaohsiung, 807 Taiwan; 11 Graduate Institute of Clinical Medicine, College of Medicine, Kaohsiung Medical University, Kaohsiung, Taiwan; 12 Center for Biomarkers and Biotech Drugs, Kaohsiung Medical University, Kaohsiung, Taiwan; 13 College of Pharmacy, Taipei Medical University, Taipei, Taiwan

**Keywords:** Upper rectal cancer, Middle/lower rectal cancer, Neoadjuvant chemoradiation, FOLFOX, CCRT

## Abstract

**Background:**

We aimed to explore the efficacy and safety profile of preoperative neoadjuvant chemoradiation (NACRT) in locally advanced rectal cancer (LARC) in upper rectum versus middle/lower rectum.

**Methods:**

The study included 173 patients with stage II or III (T2-4b, N0-2b) LARC who underwent NACRT followed by total mesorectal excision (TME) between January 2011 and October 2016. Cox regression, log-rank test, and Kaplan–Meier curves were calculated.

**Results:**

Among the 173 patients, 58 had lesions in the upper rectum and 115 patients had lesions in middle/lower rectum. In a median follow-up of 35 months (range, 6–73 months), the 5-year disease-free survival (DFS) and overall survival (OS) were 84% and 88% for the patients with upper rectal cancer and 77% and 68% for those with middle/lower rectal cancer (*P* = 0.251 and *P* = 0.058, respectively). The 5-year DFS (*P* = 0.012) and OS (*P* = 0.003) were better in the NACRT responders [tumor regression grade (TRG) 0 or 1] compared with nonresponders (TRG 2 or 3). The independent prognostic factor of favorable response to NACRT was the FOLFOX regimen (*P* = 0.004).

**Conclusions:**

Patients with LARC in the upper rectum who underwent NACRT followed by TME had similar DFS and a trend toward longer OS, compared with those who had middle/lower rectal lesions. Furthermore, FOLFOX may yield superior results than fluoropyrimidine based regimen during NACRT. NACRT might be an alternative option for patients with LARC in the upper rectum as it has a favorable pathological complete response rate and comparable clinical outcomes when compared with patients with LARC in middle/lower rectum.

## Background

Most patients with rectal cancer often encounter multimodality treatment including surgery, radiotherapy (RT) and chemotherapy [1]. The 5-year relative survival rate improved from 45% from 1975 to 1977 to 70% from 2006 to 2012 for patients with regional-stage rectal cancer [2]. This improvement reflects advances in treatment such as preoperative neoadjuvant chemoradiation (NACRT) for locally advanced rectal cancer (LARC) [2]. By utilizing the National Cancer Data Base, researchers studied a total of 66,197 patients who had been diagnosed with stage II to III rectal adenocarcinoma and treated between 2004 and 2012. The 5-year overall survival (OS) rates for patients treated with NACRT followed by surgery, surgery and adjuvant chemoradiotherapy (CRT), surgery alone, and definitive CRT were 72.4%, 70.9%, 44.9%, and 48.8%, respectively [3]. The trimodality therapy of NACRT followed by surgery is associated with the best outcomes. The patients who received NACRT had improved OS (hazard ratio, 0.77; *P* < 0.01) compared with those who received neoadjuvant multiagent chemotherapy regimens without RT. This effect was confirmed by a propensity score matching analysis (hazard ratio, 0.72; *P* = 0.01) [4].

According to the data compiled by the European Society for Medical Oncology (ESMO) Guidelines Committee, preoperative RT or CRT reduces the rate of local recurrence without improving OS for middle/lower stage II/III rectal cancers [5, 6]. Considering the heterogeneity among literature reviews, the ESMO panelists did not recommend preoperative NACRT for upper rectal ancers (> 12 cm from the anal verge) above the peritoneal reflection [7–13]. There is significant unmet clinical need for this group of patients.

As for chemotherapy regimen, the combination of 5-FU and oxaliplatin (FOLFOX) was shown to improve survival and prolong time to progression in previously untreated metastatic colon and rectal cancer when compared with fluorouracil plus leucovorin or irinotecan [14]. When adding oxaliplatin to NACRT, results have not been conclusive in terms of survival, although improved pathological complete response rate (pCR) was found in some studies [15–19].

Thus, we investigated patients with LARC in the upper versus middle/lower rectum who underwent preoperative treatment with FOLFOX- or fluoropyrimidine-based NACRT and present their pCR, NACRT-related toxicity, prognostic factors, and oncological outcomes.

## Materials and methods

### Patients

We analyzed a series of 173 consecutive patients with pathologically proven rectal adenocarcinoma (T2-4b, N0-2b) between January 2011 and October 2016. All the patients had no metastasis at diagnosis and received preoperative NACRT followed by total mesorectal excision (TME). The exclusion criteria for this study were local excision of tumor, a history of prior pelvic irradiation, and a history of malignancies other than rectal cancer. The present study was approved by the Institutional Review Board (IRB) of Kaohsiung Medical University Hospital (IRB approval number: KMUHIRB-E(II)-20170179).

All the patients underwent pretreatment workups comprising a physical examination, a history review, a colonoscopy, a tumor biopsy, chest radiography, abdominal computed tomography (CT), pelvic magnetic resonance imaging (MRI), a serum carcinoembryonic antigen (CEA) test, and routine laboratory studies. The median follow-up was 35 months (range, 6–73 months). As measured by rigid sigmoidoscopy or pelvic CT scan, the distal extension of all tumors was no more than 15 cm from the anal margin and categorized as lower (up to 5 cm), middle (from > 5 to 10 cm) or upper (from > 10 to 15 cm). The tumor stage was classified according to the seventh edition of the American Joint Committee on Cancer (AJCC) Cancer Staging Manual and Handbook [20].

### Ethics approval statement

The present study (KMUHIRB-E(II)-20,170,179) was conducted under compliance of the IRB regulations of Kaohsiung Medical University Hospital. All patients provided written informed consent prior to NACRT and TME. Patient information was anonymized and de-identified before analysis. All data were analyzed anonymously and retrospectively. The need for consent was waived by the IRB for retrospective chart reviews.

### Chemotherapy

During NACRT, 94 patients were treated with a fluoropyrimidine-based regimen that comprised one of the following: (i) 5-FU (350 mg/m^2^, intravenous bolus) and leucovorin (20 mg/m^2^, intravenous bolus) on days 1 through 5 and days 21 through 25 fractions of RT, once every 2 weeks; or (ii) six cycles of capecitabine 850 mg/m^2^ twice daily for 14 days, followed by 7 days of rest after each cycle. Moreover, 79 patients were prescribed with FOLFOX biweekly, concurrent with radiotherapy. Oxaliplatin (85 mg/m^2^) on the first day, folinic acid (400 mg/m^2^), and a 46-h infusion of 5-FU (2800 mg/m^2^) were given.

### Radiotherapy

All patients were asked to void and then drink 8 oz. of water half an hour before CT simulation and each fraction of RT. CT-based treatment planning was performed to identify bowel and bladder volumes, and minimize the exposure of these organs. Each patient was simulated in the supine position in a customized thermoplastic immobilization cast.

Three-dimensional conventional radiotherapy (3D-CRT) was delivered using a 2100 C/D linear accelerator (Varian Medical Systems, Palo Alto, CA). For the 3D-CRT plan, we used a three-field technique with two opposed lateral fields and one posterior–anterior field with wedges and photon energy of 10 MV. The radiation portal fields were designed as: (i) superior border: L5–S1 interspace, (ii) inferior border: 3–4 cm below the primary tumor, (iii) lateral border: 1.5 cm outside the true bony pelvis, (iv) posterior margin: 1.5 cm behind the anterior bony sacral margin, and (v) anterior border: posterior border of the symphysis pubis. Pelvic radiotherapy consisted of 45 Gy in 25 fractions over a period of 5 weeks; followed by a boost dose of 5.4 Gy administered in 3 fractions to the primary tumor and involved nodes by two lateral fields. The dose specification for 3D-CRT is to encompass the planning target volume (PTV) in all directions with the 95% isodose line. The volume receiving more than 110% of the dose prescribed to the PTV was minimized. The reference point was selected either in the central part of PTV or at the intersection of the beam axes by International Commission on Radiation Units and Measurements (ICRU) (Report 50 and 62).

The image-guided intensity-modulated radiotherapy (IG-IMRT) plans were generated either with a Hi-Art helical tomotherapy unit, version 2.2.4.1 (TomoTherapy, Inc., Madison, WI), or Eclipse, version 8.6 (Varian Medical Systems Inc., Palo Alto, USA). TomoTherapy combines a rotational IMRT with a translational movement of the couch. A fixed-jaw mode with a field width of 2.5 or 5 cm was used for treatment planning. The pitch varied from 0.215 to 0.287. The modulation factor ranged from 2 to 3, depending on the homogeneity and conformity. The gross tumor volume encompassed rectal tumors and clustered lymph nodes or lymph nodes with a diameter greater than 1 cm. The clinical target volume (CTV) included the primary tumor, the mesorectum, the sacral canal, and the perirectal, presacral, hypogastric, obturator, and internal iliac lymphatic drainage. A superior, an inferior, and a radial margin of 5 to 7 mm outside the CTV were added to form the PTV.

In the IG-IMRT group, the tumor and boost beams were combined in one integrated treatment plan; thus, these patients were treated with the same plan for each fraction throughout the entire course of RT. Fractionation schemes were 25 daily fractions of 1.8 Gy to the pelvis and 2 Gy to the rectal tumor and involved nodes. Optimization was addressed to reduce the dose for the bowel, bladder, and femoral heads. These constraints were also applied to the IMRT treatment plans on Varian and comprised beams with multileaf collimator shielding conformal to the PTV. The goal was to encompass the PTV in all directions with the 95% isodose line. Volumes receiving more than 110% of the dose prescribed to the PTV were minimized. Volumetric arc therapy was used when suitable. The IMRT plans were reviewed using ICRU 83 recommendations. Before each fraction of RT, patients were repositioned according to image guidance through a megavoltage or cone beam CT, which was coregistered with a planning kilovoltage CT. A dose of 50 Gy was prescribed to the PTV_50_ (tumor and enlarged nodes) and 45 Gy to the PTV_45_ (pelvic nodal area) by simultaneous integrated boost scheme in the IG-IMRT group. All dose schedules were given 5 days per week.

### Surgery

All 173 patients had TME with a median of 10 weeks (range, 6–22) after completion of the NACRT. TME was performed for each patient so that all the mesorectal fat and the encompassing lymph nodes were meticulously excised [17, 21]. Twenty-five patients with clinical T4 classification had extended adjacent visceral resection. We performed anal sphincter-sparing surgery when applicable, with primary anastomosis and/or temporary diverting colostomies.

### Toxicity

The surgeons and radiation oncologists recorded acute toxicities according to the Common Terminology Criteria of Adverse Events (CTCAE), version 4.03 (http://ctep.cancer.gov/reporting/ctc.html). The scoring was performed as the patients underwent the treatment administered by the attending physicians and once per week during NACRT. As for the skin care management strategy, topical silver sulfadiazine (SSD) was prescribed for acute radiation dermatitis grade 2 or higher. Those patients who required it received SSD cream 1%, once per day, preferably after a shower or bath. When chemo agents produced severe side effects, dose reduction and/or the temporary suspension of medication were exerted.

### Evaluation

We assessed efficacy by using the pCR rate and tumor regression grade (TRG). We defined patients with TRG 0 or 1 as NACRT responders and those with TRG 2 or 3 as nonresponders. A pCR was defined as the absence of any viable residual tumor cell in the resected primary tumor and adjacent lymph nodes (ypT0N0) after NACRT. We then compared the clinical stage with the pathological stage to determine the down-staging rate. The TRG was recorded by the AJCC system [22]. A circumferential resection margin (CRM) of less than 1 mm was defined as a positive CRM.

The primary end points were disease-free survival (DFS) and OS. The secondary end points were acute toxicities during NACRT and the pCR rate after the preoperative NACRT. In general, the patients were observed with a standardized follow-up every 3 months after therapy for the first year and every 6 months thereafter. The length of follow-up was defined as the time from NACRT to the date of death or last follow-up. Local failure was defined as any disease recurrence within the pelvis. Any failure outside these regions was classified as a distant metastasis. Recurrence was confirmed pathologically by surgical resection, biopsy, or cytology, and/or radiological findings, which increased in size over time. Distant metastasis was recorded mostly according to chest radiography, abdominal ultrasonography, CT scan, magnetic resonance imaging, or technetium-99 bone scintigraphy.

### Statistical analysis

The data set was stratified and outcomes were compared by t test or chi-squared test. Univariate analyses and a multivariate Cox proportional hazards regression were used to examine the following characteristics and their potential association with the response to NACRT: age, gender, RT technique, and the tumor’s distance from the anal verge, clinical tumor depth, clinical lymph node metastasis, and different chemotherapy regimens.

OS was defined as the time from the date of primary treatment to the date of death from any cause or until the date of the last follow-up. DFS was defined as the time from the date of primary treatment to the date of diagnosis for recurrence or metastatic disease or to the date of the last follow-up. Locoregional failure-free survival (LFFS) was defined as the time from the date of primary treatment to the date of diagnosis for recurrence or to the date of the last follow-up. Distant metastasis failure-free survival (DMFS) was defined as the time from the date of primary treatment to the date of diagnosis for metastatic disease or to the date of the last follow-up.

OS, DFS, LFFS, and DMFS were assessed by Kaplan–Meier methods and the log-rank test was used to compare time-to-event distributions. Estimated risks of death were calculated using hazard ratios (HR) with 95% confidence intervals (CIs). The level of statistical significance was set at *P* < 0.05; all reported *P* values were two-tailed. The analyses were performed using the SPSS software package, version 19.0 for Windows (SPSS, Chicago, IL, USA).

## Results

**Fig. 1:**
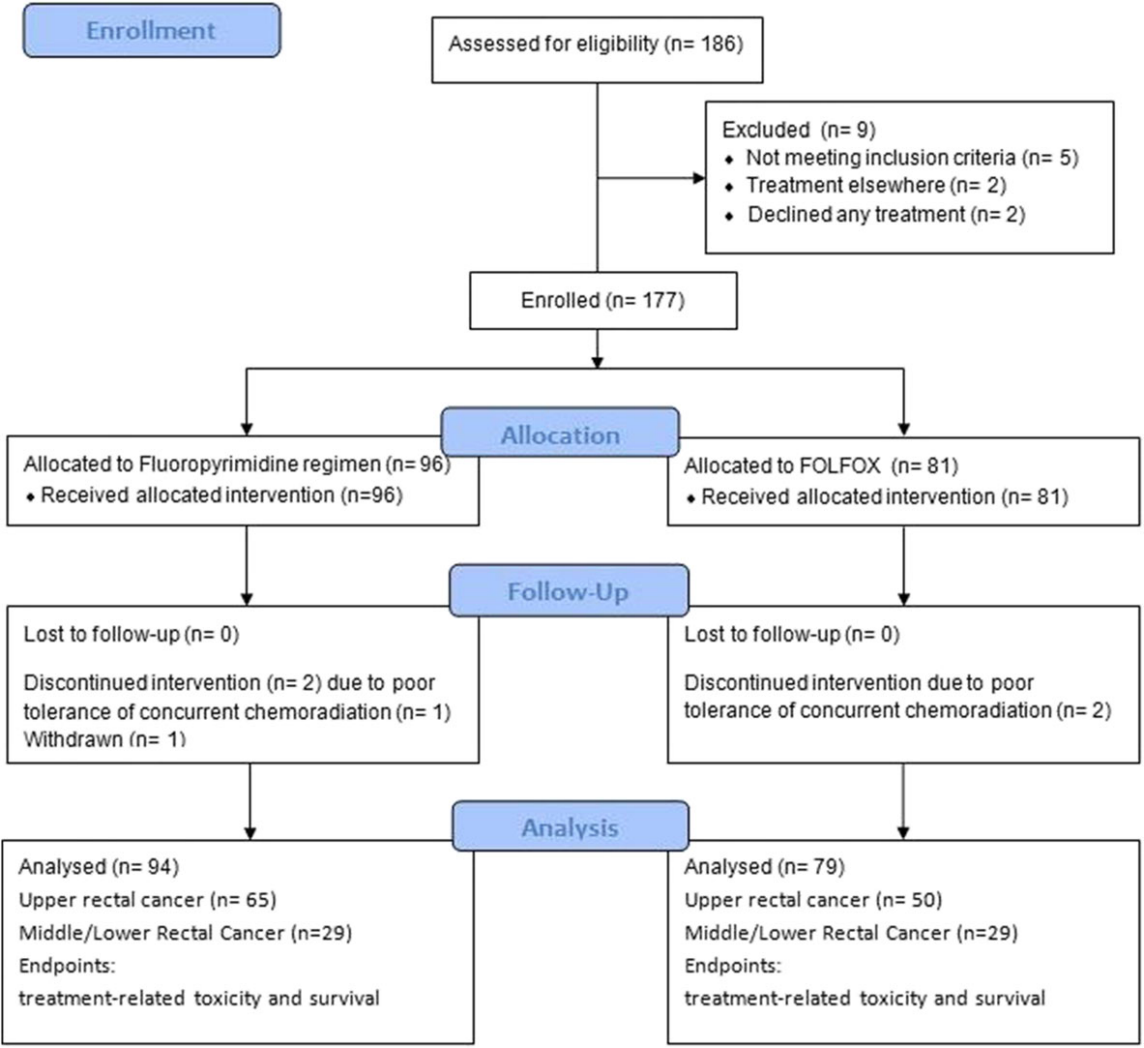
Patient enrollement flow diagram

Figure 1 is the CONSORT diagram. The median age of this retrospective cohort was 61 years (range, 34–93 years). The male-to-female ratio was 2:1. Table 1 summarizes the clinical characteristics of the 173 patients, divided by the location of their tumors into the middle/lower rectum group and upper rectum group. The methods of treatment were as follows: 54.3% of patients received fluoropyrimidine-based chemotherapy; 45.7% received FOLFOX, and 74.6% underwent IG-IMRT. The median RT dose was 50 Gy (range, 45–54 Gy). No significant differences were observed in terms of age, gender, clinical T classification, clinical N classification, clinical stage, pretreatment CEA level, chemotherapy regimen, RT technique, and median RT dose, with or without postoperative adjuvant chemotherapy and follow-up time between upper rectal group and middle/lower rectal group (all *P* > 0.05; Table 1).

**Table 1:** Patient characteristics in upper rectum and middle/lower rectum groups

Characteristics	Total		Upper		Middle/Lower		*P*-value
	N	(%)	*n* = 58	(%)	*n* = 115	(%)	
Age, median, year (range)	61(34–93)		63.5(34–87)		61(34–93)		0.268
Age							
< 65	109	63	35	60.3	74	64.3	0.607
> = 65	64	37	23	39.7	41	35.7	
Gender							
Male	115	66.5	33	56.9	82	71.3	0.063
Female	58	33.5	25	43.1	33	28.7	
Clinical tumor depth							
T2	11	6.4	3	5.2	8	7	0.108
T3	137	79.2	41	70.7	96	83.5	
T4a	13	7.5	10	17.2	3	2.6	
T4b	12	6.9	4	6.9	8	7	
Clinical lymph node metastasis							
N0	28	16.2	7	12.1	21	18.3	0.611
N1	106	61.3	40	69	66	57.4	
N2a	28	16.2	10	17.2	18	15.7	
N2b	11	6.4	1	1.7	10	8.7	
Clinical stage							
2	28	16.2	7	12.1	21	18.3	0.297
3	145	83.8	51	87.9	94	81.3	
Pretreatment CEA (ng/ml)							
< = 5	110	63.6	35	60.3	75	65.2	0.53
> 5	63	36.4	23	39.7	40	34.8	
Concurrent chemotherapy							
Fluoropyrimidine	94	54.3	29	50	65	56.5	0.416
FOLFOX	79	45.7	29	50	50	43.5	
RT technique							
3DCRT^a^	44	25.4	15	25.9	29	25.2	0.927
IG-IMRT^b^	129	74.6	43	74.1	86	74.8	
Median RT dose, Gy (range)							
5000 (4500–5400)			5000 (4500–5040)		5000 (4500–5400)		0.454
Adjuvant chemotherapy							
Yes	122	70.5	44	75.9	78	67.8	0.274
No	51	29.5	14	24.1	37	32.2	
Median follow-up, month (range)							
	35(6–73)		32(7–71)		36(6–73)		0.992

^a^three-dimensional conventional radiotherapy; ^b^image guided intensity modulated radiotherapy

Table 2 summarizes the pathological characteristics of the 173 patients. The pCR was 22.5%, and the negative CRM rate was 96.5%. The down-staging rates of the T and N classifications were 64.7% and 67.6%, respectively. Additionally, 19.1% of patients had perineural invasion, 12.1% had lymphovascular invasion, and 88.4% had moderate tumor differentiation. No significant differences in pathological tumor depth, nodal classification, pCR, median number of resected lymph nodes, TRG, CRM, perineural invasion, lymphovascular invasion, tumor differentiation, and T/N down-staging rates were observed between the upper and middle/lower groups (all *P* > 0.05; Table 2).

**Table 2:** Patient characteristics in upper rectum and middle/lower rectum groups

Characteristics	Total		Upper		Middle/Lower		*P*-value
	N	(%)	*n* = 58	(%)	*n* = 115	(%)	
Pathologic tumor depth							
ypT0	39	22.5	11	19	28	24.3	0.076
ypT1	16	9.2	4	6.9	12	10.4	
ypT2	43	24.9	12	20.7	31	27	
ypT3	72	41.6	29	50	43	37.4	
ypT4a	1	0.6	0	0	1	0.9	
ypT4b	2	1.2	2	3.4	0	0	
Pathologic lymph node metastasis							
ypN0	132	76.3	47	81	85	73.9	0.105
ypN1a	17	9.8	6	10.3	11	9.6	
ypN1b	10	5.8	3	5.2	7	6.1	
ypN1c	5	2.9	1	1.7	4	3.5	
ypN2a	6	3.5	1	1.7	5	4.3	
ypN2b	3	1.7	0	0	3	2.6	
Pathologic complete response							
Yes	39	22.5	11	19	28	24.3	0.424
No	134	77.5	47	81	87	75.7	
Median number of resected nodes	11 (0–25)		11(5–25)		11 (0–23)		0.91
Median number of involved nodes	0(0–9)		0 (0–4)		0(0–9)		
Tumor regression grade							
0	39	22.5	11	19	28	24.3	0.371
1	58	33.5	27	46.6	31	27	
2	52	30.1	14	24.1	38	33	
3	24	13.9	6	10.3	18	15.7	
Circumferential resection margin							
Negative	167	96.5	55	94.8	112	97.4	0.404
Positive	6	3.5	3	5.2	3	2.6	
Perineural invasion							
Negative	140	80.9	47	81	93	80.9	0.979
Positive	33	19.1	11	19	22	19.1	
Lymphovascular invasion							
Negative	152	87.9	52	89.7	100	87	0.608
Positive	21	12.1	6	10.3	15	13.413	
Tumor differentiation							
Well	13	7.5	4	6.9	9	7.8	0.996
Moderately	153	88.4	52	89.7	101	87.8	
Poorly	7	4	2	3.4	5	4.4	
Pathologic T stage							
Downstaging	112	64.7	34	58.6	78	67.8	0.462
Stable	55	31.8	23	39.7	32	27.8	
Progressive	6	3.5	1	1.7	5	4.3	
Pathologic N stage							
Downstaging	117	67.6	41	70.7	76	66.1	0.21
Stable	48	27.7	17	29.3	31	26.9	
Progressive	8	4.6	0	0	8	7	
Pathologic TN stage							
Downstaging	142	82.1	50	86.2	92	80	0.115
Stable	23	13.3	8	13.8	15	13	
Progressive	8	4.6	0	0	8	7	

As shown in Table 3, IG-IMRT was associated with less acute urological toxicity compared with 3D-CRT in middle/lower group (*P* = 0.014). No acute urological toxicity was observed in the patients with upper rectal cancer. No significant differences in leukocyte counts, hemoglobin levels, dermatological and gastrointestinal side effects, and a prolonged RT course of more than 40 days between the upper rectal and middle/lower groups (all *P* > 0.05) were observed. No new safety concerns were identified in the current study (Table 3).

**Table 3:** Comparision of toxicity and treatment breaks in 3DCRT and IG-IMRT groups

Acute toxicity			3DCRT^a^		IG-IMRT^b^		Intra-goup	Inter-group
			*n* = 44	(%)	*n* = 129	(%)	*P*-value	*P*-value
Skin	Middle/Lower rectum	Grade 0	17	58.6	57	66.3	0.204	0.103
		Grade 1	8	27.6	24	27.9		
		Grade 2	2	6.9	3	3.5		
		Grade 3	2	6.9	2	2.3		
	Upper rectum	Grade 0	9	60	33	76.7	0.216	
		Grade 1	6	40	10	23.3		
		Grade 2	0	0	0	0		
		Grade 3	0	0	0	0		
GI^c^	Middle/Lower rectum	Grade 0	13	44.8	48	55.8	0.284	0.071
		Grade 1	8	27.6	21	24.4		
		Grade 2	4	13.8	9	10.5		
		Grade 3	4	13.8	8	9.3		
	Upper rectum	Grade 0	6	35.7	25	58.1	0.099	
		Grade 1	4	28.6	12	27.9		
		Grade 2	3	21.4	4	9.3		
		Grade 3	2	14.3	2	4.7		
GU^d^	Middle/Lower rectum	Grade 0	24	82.8	79	91.9	0.014	0.016
		Grade 1	2	6.9	7	8.1		
		Grade 2	1	3.4	0	0		
		Grade 3	2	6.9	0	0		
	Upper rectum	Grade 0	15	100	43	100	–	
		Grade 1	0	0	0	0		
		Grade 2	0	0	0	0		
		Grade 3	0	0	0	0		
Leukocytosis	Middle/Lower rectum	Grade 0–2	29	100	86	100	–	–
		Grade 3	0	0	0	0		
	Upper rectum	Grade 0–2	15	100	43	100	–	
		Grade 3	0	0	0	0		
Hemoglobin	Middle/Lower rectum	Grade 0	7	24.1	27	31.4	0.522	0.448
		Grade 1	13	44.8	37	43		
		Grade 2	8	27.6	18	20.9		
		Grade 3	1	3.5	4	4.7		
	Upper rectum	Grade 0	3	20	10	23.3	0.593	
		Grade 1	5	33.3	20	46.5		
		Grade 2	6	40	8	18.6		
		Grade 3	1	6.7	5	11.6		
RT^e^ ≥ 40 days	Middle/Lower rectum	Yes	2	6.9	5	5.8	0.834	0.733
		No	27	93.1	81	94.2		
	Upper rectum	Yes	1	6.7	2	4.7	0.764	
		No	14	93.3	41	95.3		

^a^Three-dimensional conventional radiotherapy; ^b^image guided intensity modulated radiotherapy; ^c^Gastrointestinal tract; ^d^genitourinary tract; ^e^RT: radiotherapy

### Survival

Table 4 outlines the association between the NACRT responders and nonresponders by univariate and multivariate analyses. Response was significantly worse with a fluoropyrimidine-based regimen compared with that with FOLFOX (*P* = 0.004, OR = 2.604; 95% CI, 1.346–5.038). The tumor location of upper versus middle/lower rectum did not affect the NACRT response (*P* = 0.087, OR = 1.847; 95% CI, 0.915–3.725). Age, gender, RT technique, and clinical T and N classifications were not independent prognostic factors (all *P* > 0.05; Table 4).

**Table 4:** Prognostic factors analysis for neoadjuvant chemoradiation responder

Characteristics			No responder		Responder		Univariate	Multivariate		
	Total		TRG2 + TRG3^a^		TRG0 + TRG1^a^		*P*-value	*P*-value	OR	95%CI
	N	(%)	*n* = 76	(%)	*n* = 97	(%)				
Age										
< 65	109	63	50	65.8	59	60.8	0.502	0.439	0.77	0.397–1.493
> = 65	64	37	26	34.2	38	39.2				
Gender										
Male	115	66.5	54	71.1	61	62.9	0.259	0.223	0.653	0.329–1.297
Female	58	37.1	22	28.9	36	37.1				
RT^b^ technique										
3DCRT^c^	44	25.4	21	27.6	23	23.7	0.557	0.934	1.031	0.496–2.145
IG-IMRT^d^	129	74.6	55	72.4	74	76.3				
Location (rectum)										
Middle/Lower	115	66.5	56	73.7	59	60.8	0.075	0.087	1.847	0.915–3.725
Upper	58	33.5	20	26.3	38	39.2				
Clinical tumor depth										
T4	25	14.5	11	14.5	14	14.4	0.994	0.292	1.676	0.642–4.376
T2–3	148	85.5	65	85.5	83	85.6				
Clinical lymph node metastasis										
N1–2	145	83.8	66	86.8	79	81.4	0.339	0.137	1.972	0.805–4.832
N0	28	16.2	10	13.2	18	18.6				
Concurrent chemotherapy										
Fluoropyrimidine	94	54.3	50	65.8	44	45.4	0.007	0.004	2.604	1.346–5.038
FOLFOX	79	45.7	26	34.2	53	54.6				

^a^Tumor regression grade; ^b^radiotherapy; ^c^three-dimensional conventional radiotherapy; ^d^image guided intensity modulated radiotherapy

**Fig. 2:**
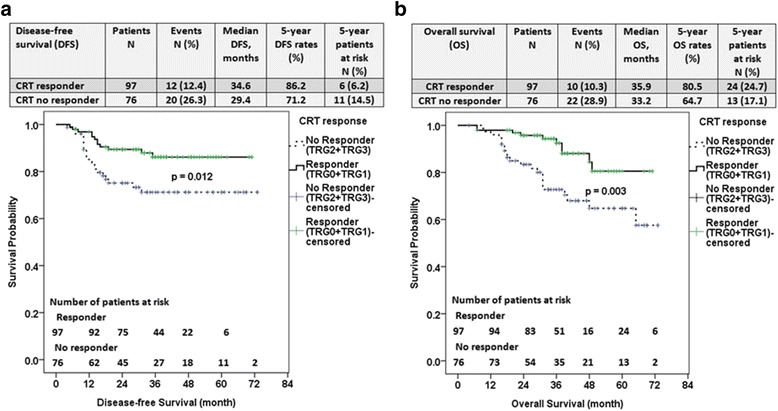
Disease-free survival **(**DFS) and overall survival (OS) between neoadjuvant chemoradiation (NACRT) responders and nonresponders. Kaplan–Meier curves demonstrated DFS (**a**) and OS (**b**) between NACRT responders and nonresponders. Among 173 patients, 97 patients were responders and 76 patients were nonresponders. At a mean follow-up time of 35 months (range, 6–73), significant differences in the 5-year DFS (*P* = 0.012) and 5-year OS rates (*P* = 0.003) were observed between the two groups

In Fig. 2a and b, the Kaplan–Meier curves demonstrated the DFS and OS between the NACRT responders and nonresponders. Among the 173 patients, 97 patients were responders and 76 patients were nonresponders. At a median follow-up time of 35 months (range, 6–73), significant differences were observed in the 5-year DFS rates (*P* = 0.012) and 5-year OS rates (*P* = 0.003) between the two groups. The median DFS and OS were 34.6 months versus 35.9 months for the NACRT responders and 29.4 months versus 33.2 months for the nonresponders (Fig. 2a and b).

**Fig. 3:**
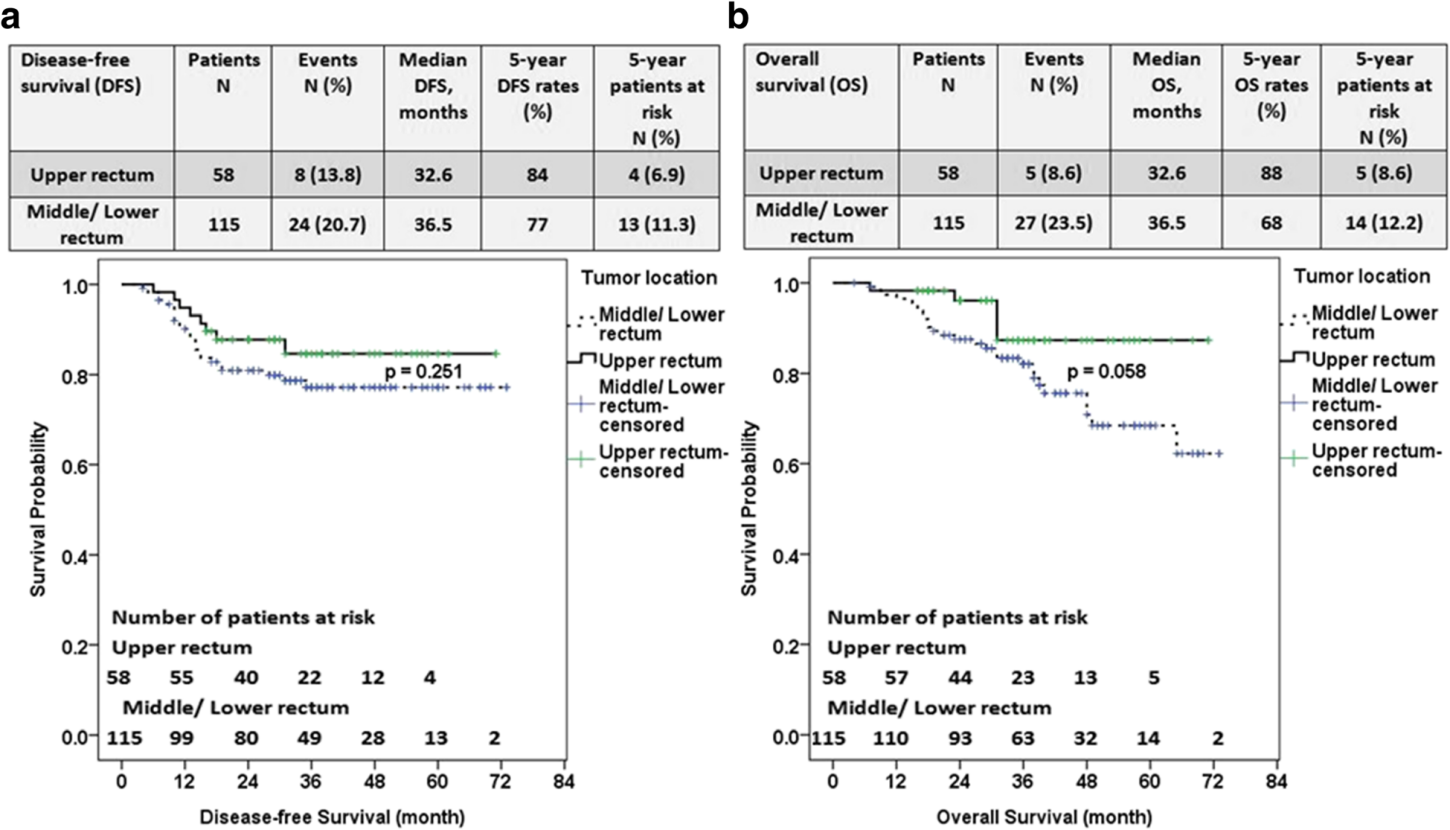
Disease-free survival (DFS) (**a**) and overall survival (OS) (**b**) between upper rectal versus middle/lower rectal cancer patients receiving neoadjuvant chemoradiation therapy: 58 patients had lesions in the upper rectum and 115 patients had lesions in the middle/lower rectum. The 5-year DFS and 5-year OS rates were 84% and 88% for the patients with upper rectal cancer and 77% and 68% for those with middle/lower rectal cancer (*P* = 0.251 and *P* = 0.058, respectively)

In Fig. 3a and b, 58 patients had lesions in the upper rectum and 115 patients had middle/lower rectal tumors. The 5-year DFS and 5-year OS rates were 84% and 88% for the patients with upper rectal cancer and 77% and 68% for those with middle or lower rectal cancer (*P* = 0.251 and *P* = 0.058, respectively). The local recurrence rates between the patients with upper versus middle/lower rectal cancer were 8.6% versus 9.6%, and the distant metastasis rates were 6.9% versus 13% between upper versus middle/lower rectal cancer patients (Fig. 3a and b).

**Fig. 4:**
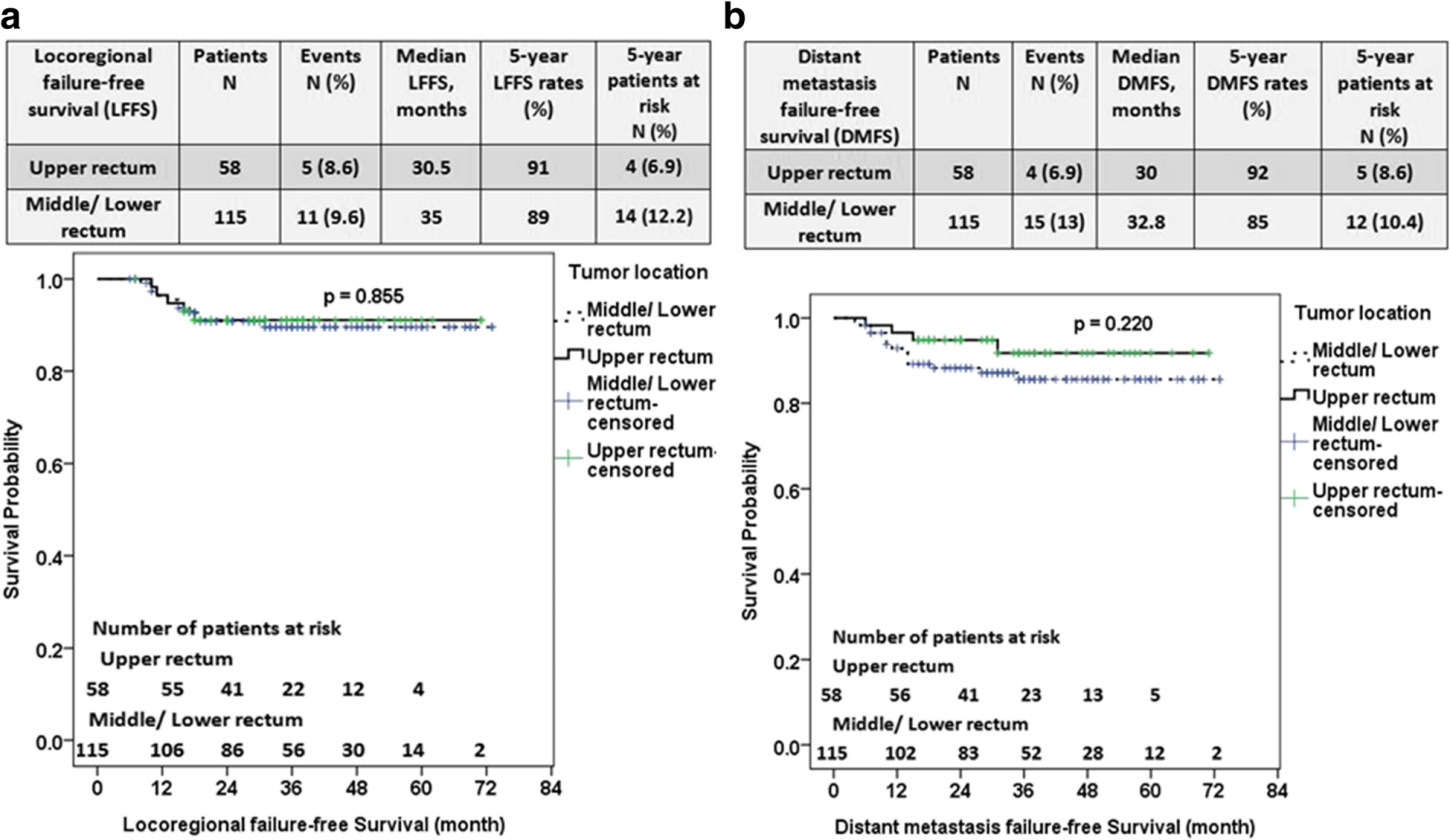
Locoregional failure-free survival (LFFS) and distant metastasis failure-free survival (DMFS) between upper rectal versus middle/lower rectal cancer patients receiving neoadjuvant chemoradiation therapy. The local recurrence rate between upper versus middle/lower rectal cancer patients were 8.6% versus 9.6% (**a**), and the distant metastasis rate were 6.9% versus 13% between upper versus middle/lower rectal cancer patients (**b**). The 5-year LFFS and 5-year DMFS rates were 91% and 92% for those with upper rectal cancer and 89% and 85% for those with middle/lower rectal cancer (*P* = 0.855 and *P* = 0.220, respectively)

In Fig. 4a and b, the 5-year LFFS and 5-year DMFS rates were 91% and 92% for the patients with upper rectal cancer and 89% and 85% for those with middle/lower rectal cancer (*P* = 0.855 and *P* = 0.220, respectively).

## Discussion

The present study demonstrated the long-term survival of NACRT for patients with transmural and/or node-positive disease. Because the risk of local recurrence decreases with upper rectal tumors and the advantage of sphincter preservation is not pertinent, some studies had proposed up-front surgery [7, 8, 10, 11, 13]. In a multicenter randomized controlled trial from 1987 to 1993, Kaser and colleagues evaluated 725 R0-resected colorectal cancer patients without neoadjuvant or adjuvant RT or TME. The 5-year DFS and 5-year OS rates were 54% (95%CI, 0.47–0.60) and 64% (95%CI, 0.57–0.71) in their patients with cancers in the lower two-thirds of the rectum (11 cm or less from anal verge), whereas the 5-year DFS and 5-year OS rates were 68% (95%CI, 0.60–0.75) and 79% (95%CI, 0.71–0.85) in those with the upper-third of the rectum and recto–sigmoid junction (> 11–20 cm from anal verge), respectively [23].

In our study of 173 patients with NACRT plus TME, the 5-year DFS rate and 5-year OS rate were 84% and 88% for the patients with upper rectal cancer and 77% and 68% for those with middle/lower rectal cancer (*P* = 0.251 and *P* = 0.058, respectively). Marinello et al. investigated 147 patients with upper rectal cancers who underwent partial mesorectal excision; among them only 5.6% received NACRT. Their 5-year actuarial DFS was 82.0% in a single-institution, retrospective study [24].

Roh et al. identified 244 patients with clinical T3, T4, or node-positive rectal cancer who were randomly assigned to preoperative or postoperative chemoradiotherapy from the NSABP R-03 (National Surgical Adjuvant Breast and Bowel Project R-03) trial [25]. They found NACRT significantly increased DFS and increased a trend toward improved OS. The 5-year DFS for the NACRT patients was 64.7% versus 53.4% for the postoperative patients (*P* = 0.011); however, the 5-year OS for the NACRT patients was 74.5% versus 65.6% for the postoperative patients (*P* = 0.065) [25]. A pCR was achieved in 15% of NACRT patients, whereas in our cohort study the pCR rate was 22.5%.

A multidisciplinary team of European cancer experts achieved consensus on risk-adapted treatment in that patients with cT4 tumors falling back into the pelvis might benefit from NACRT [5]. Yet, they do not recommend this treatment paradigm be applied uniformly to all patients irrespective of tumor location. Jorgren et al. conducted a population-based survey from the Swedish rectal cancer registry [9]. They assessed 4153 patients and concluded preoperative RT should be considered for upper rectal cancer. Rosenberg et al. studied 499 patients and documented that tumor distance from the anal verge was an independent prognostic parameter (*P* = 0.036), with an increased risk of cause-specific death for rectal cancers of the upper third (hazard ratio, 1.87; *P* = 0.007) and middle third (hazard ratio, 1.43; *P* = 0.022) compared with sigmoid cancers [12]. McCarthy et al. analyzed 6 randomised controlled trials and found a reduction in local recurrence in the NACRT group in comparison to the preoperative RT group (OR = 0.56, 95%CI 0.42–0.75, *P* < 0.0001), yet the results for overall survival were (OR = 1.01 95%CI 0.85–1.20, *P* = 0.88) for these patients with T3–4, node positive (locally advanced) rectal cancer [26]. Abdel et al. studied 1680 patients with locally advanced rectal cancer and found NACRT increased median overall survival of 42.7 compared to 37.3 and 26.6 months for neoadjuvant chemotherapy and no neoadjuvant therapy, respectively (*P* < 0.0001) [27]. The evidence is growing that NACRT may alter prognosis for patients with rectal cancer. Neoadjuvant treatment did impact survival. Our present study corroborates this effect.

Preoperative RT improves local control in patients with rectal cancer, particularly NACRT. The question if the use of more effective chemotherapy improves OS remained unanswered [28]. As for oxaliplatin and its relation to survival, Allegra and colleagues investigated 1608 randomized patients and reported no statistically significant difference between regimens using 5-FU versus capecitabine in 3-year local–regional tumor event rates (11.2% vs 11.8%), 5-year DFS (66.4% vs 67.7%), or 5-year OS (79.9% vs 80.8%); likewise, no statistically significant difference was found for oxaliplatin versus no oxaliplatin for the three endpoints of local–regional events, DFS, and OS (11.2% vs 12.1%, 69.2% vs 64.2%, and 81.3% vs 79.0%) [29].

In the present study, the authors compared the long-term clinical outcomes in a cohort of 173 patients who underwent NACRT followed by TME. The authors found no significant difference in DFS or OS between patients with upper or middle/lower rectal lesions. In this study, the NACRT responders (TRG 0–1) had a higher DFS and OS than the nonresponders (TRG 2–3) (86.2% vs 71.2% and 80.5% vs 64.7%, respectively (Fig. 2a and b). Additionally, FOLFOX significantly enhanced response rate when compared with a fluoropyrimidine-based regimen. Dolinsky et al. found that patients receiving 5 FU/oxaliplatin/RT had a high pCR rate compared with that with 5-FU/RT [15]. Huang et al. reported a pCR rate of 31.6% with the sphincter preservation rate of 92.2% (39/42) in patients with tumors located less than 5 cm from the anal verge [17]. Their 2-year overall and disease-free survivals were 94% and 87.4%, respectively after NACRT with FOLFOX regimen [17]. However, adding oxaliplatin did not improve surgical outcomes but added significant toxicity according to the preliminary result from National Surgical Adjuvant Breast and Bowel Project trial R-04 [18]. They have not performed definitive analysis of local tumor control, DFS, and OS yet [18]. On the other hand, the German CAO/ARO/AIO-04 study, a multicentre, open-label, randomised, phase 3 trial, concluded that adding oxaliplatin to fluorouracil-based NACRT and adjuvant chemotherapy significantly improved DFS of patients with clinically staged cT3–4 or cN1–2 rectal cancer compared with fluorouracil-based regimen (based on CAO/ARO/AIO-94) [19]. We need to see FOLFOX improve survival in more studies to make it a truly compelling new standard of care.

The limitations of the current study include the inherent biases in retrospective studies. Additionally, surgical complications were not assessed. Although the patients were not randomized by the two chemotherapy regimens or RT technique, fundamentally, similar characteristics exist between the upper and middle/lower rectum groups. In our previous study, no prominent difference in distant metastasis rate and overall survival between preoperative IG-IMRT and 3DCRT was observed [30]. In the current study, we did not focus on the merits of different RT techniques but on survival and the acute toxicity of NACRT, pCR, and TRG.

## Conclusion

This present study reported an institutional experience and found survival advantages for FOLFOX in NACRT. This treatment was generally safe and well tolerated. The patients with upper rectal cancer who underwent NACRT followed by TME had similar DFS, LFFS, and DMFS and a trend toward a longer OS, compared with those who had middle/lower lesions. Nevertheless, a prospective study with a longer follow-up time is required and continuous data collection and analyses will be pursued steadily.

## Abbreviations

3D-CRT: Three-dimensional conventional radiotherapy
5-FU: 5-fluorouracil
AJCC: American Joint Committee on Cancer
CEA: Carcinoembryonic antigen
CRC: Colorectal cancer
CRM: Circumferential resection margin
CRT: Chemoradiotherapy
CT: Computed tomography
CTCAE: Common Terminology Criteria for Adverse Events
CTV: Clinical target volume
DFS: Disease-free survival
DMFS: Distant metastasis failure-free survival
ESMO: European Society for Medical Oncology
FOLFOX: 5-fluorouracil, leucovorin, and oxaliplatin
IG-IMRT: Image-guided intensity-modulated radiotherapy
LARC: Locally advanced rectal cancer
LFFS: Locoregional failure-free survival
MRI: Magnetic resonance imaging
NACRT: Neoadjuvant chemoradiation
OS: Overall survival
pCR: Pathologic complete response
PTV: Planning target volume
RT: Radiation therapy
SSD: Silver sulfadiazine
TME: Total mesorectal excision
TRG: Tumor regression grade
